# False Ceiling Deterioration Detection and Mapping Using a Deep Learning Framework and the Teleoperated Reconfigurable ‘Falcon’ Robot

**DOI:** 10.3390/s22010262

**Published:** 2021-12-30

**Authors:** Archana Semwal, Rajesh Elara Mohan, Lee Ming Jun Melvin, Povendhan Palanisamy, Chanthini Baskar, Lim Yi, Sathian Pookkuttath, Balakrishnan Ramalingam

**Affiliations:** 1 Engineering Product Development Pillar, Singapore University of Technology and Design (SUTD), Singapore 487372, Singapore; archana_semwal@sutd.edu.sg (A.S.); rajeshelara@sutd.edu.sg (R.E.M.); melvin_lee@sutd.edu.sg (L.M.J.M.); povendhan_palanisamy@mymail.sutd.edu.sg (P.P.); yi_lim@mymail.sutd.edu.sg (L.Y.); sathian_pookkuttath@sutd.edu.sg (S.P.); 2 School of Electronics Engineering, Vellore Institute of Technology, Chennai 600127, India; chanthini.baskar@vit.ac.in

**Keywords:** defect detection, Faster R-CNN, deep learning, object detection, IoRT, inspection robot

## Abstract

Periodic inspection of false ceilings is mandatory to ensure building and human safety. Generally, false ceiling inspection includes identifying structural defects, degradation in Heating, Ventilation, and Air Conditioning (HVAC) systems, electrical wire damage, and pest infestation. Human-assisted false ceiling inspection is a laborious and risky task. This work presents a false ceiling deterioration detection and mapping framework using a deep-neural-network-based object detection algorithm and the teleoperated ‘Falcon’ robot. The object detection algorithm was trained with our custom false ceiling deterioration image dataset composed of four classes: structural defects (spalling, cracks, pitted surfaces, and water damage), degradation in HVAC systems (corrosion, molding, and pipe damage), electrical damage (frayed wires), and infestation (termites and rodents). The efficiency of the trained CNN algorithm and deterioration mapping was evaluated through various experiments and real-time field trials. The experimental results indicate that the deterioration detection and mapping results were accurate in a real false-ceiling environment and achieved an 89.53% detection accuracy.

## 1. Introduction

False ceiling inspection is one of the most required inspections for essential maintenance and repair tasks in commercial buildings. Generally, a false ceiling is built with material such as Gypsum board, Plaster of Paris, and Poly Vinyl Chloride (PVC) and used to hide ducting, messy wires, and Heating, Ventilation, and Air Conditioning (HVAC) systems. However, poor construction and the use of substandard material in false ceilings require periodic inspection to avoid deterioration. Structural defects, degradation in HVAC systems, electrical damage, and infestation are common potential building and human safety hazards. Human visual inspection is a common technique used by building maintenance companies, where trained safety inspectors will audit the environment of a false ceiling. However, deploying human visual inspection for a false ceiling environment has many practical challenges. It requires a highly skilled inspector to access a complex false ceiling environment. Workforce shortage due to safety issues and low wages is another challenge faced by false ceiling maintenance companies. These facts highlight the need for an automated, cost-effective, and exhaustive inspection of false ceilings to prevent such risks.

Hence, the aim of this research is to automate the inspection process to detect and map various deterioration factors in false ceiling environments. Further, the literature survey (Section 2) confirms a research gap between robot-assisted inspection and deep learning frameworks for false ceiling inspection and maintenance. Thus, this work presents an automated false ceiling inspection framework using a convolutional neural network trained with our false ceiling deterioration image dataset composed of four classes, structural defects (spalling, cracks, pitted surfaces, and water damage), degradation in HVAC systems (corrosion, molding, and pipe damage), electrical damage (frayed wires), and infestations (termites and rodents). Further, the inspection task is performed with the help of our in-house-developed crawl class robot, known as the ‘Falcon’, with a deterioration mapping function using Ultra-Wideband (UWB) modules. The deterioration mapping function marks the class of deteriorations with locations on a map for the inspection and maintenance of false ceilings.

This manuscript is organized as follows; after explaining the importance and contributions of the study in Section 1, Section 2 presents a literature review, and Section 3 presents an overview of the proposed system. Section 4 discusses the experimental setup and the results. Section 5 includes a discussion. Section 6 concludes.

## 2. Related Work

In recent years, various semi- or fully automated techniques have been reported in the literature for narrow and enclosed space inspections for building maintenance tasks. Here, computer vision algorithms were used for automatically detecting defects from images collected by inspection tools such as borescope cameras [1,2] and drones [3,4]. However, borescope cameras and drone-based methods have many practical difficulties when used as inspection tools in false ceiling environments. Because false ceiling environments have many protruding elements such as electrical wire networks, gas pipes, and ducts, it is also difficult to fly drones to inspect the complex environment of a false ceiling.

Robot-based inspection is a better solution than borescope cameras and drone-based inspection. It has been widely used for various narrow and enclosed space inspection applications, such as crawl space inspection [5,6], tunnel inspection [7,8], drain inspection [9,10], defect detection in glass facade buildings [11,12], and power transmission line fault detection [13]. Gary et al. proposed a q-bot inspection robot for autonomously surveying underfloor voids (floorboards, joists, vents, and pipes). It uses a mask Region Convolutional Neural Network (mask-RCNN) approach with a two-stage transferring learning method. It was able to detect with an accuracy of 80% [5]. Self-reconfigurable robot ’Mantis’ was used for crack detection, and glass facade cleaning in high-rise buildings [11,14], where a CNN-based deep learning framework with 15 layers is used for detecting cracks on glass panels. Similarly, a steel climbing robot was developed for steel infrastructure monitoring. The authors developed a steel crack detection algorithm using steel surface image-stitching and a 3D map building technique. The steel crack detection algorithm was able to achieve a success rate of 93.1% [15]. In [16], Gui et al. automated a defect detection and visualization task for airport runway inspection. The proposed novel robotic system employed a camera, Ground Penetrating Radar (GPR), and a crack detection algorithm based on images and GPR data. An F1-measure of 70% and 67% was achieved for crack detection and subsurface defect detection, respectively. In [17], Perez et al. aimed at detecting building defects (mold, deterioration, and stains) using convolutional neural networks (CNNs). The authors presented a deep-learning-based detection and localization model employing VGG-16 to extract and classify features. The tests demonstrated an overall detection accuracy of 87.50%. Xing et al., in [18], proposed a CNN-based method for workpiece surface defect detection. The authors designed a CNN model with symmetric modules for feature extraction and optimized the IoU to compute the loss function of the detection method. The average detection accuracy of the CNN on the Northeastern University-Surface Defect Database (NEU-CLS) and on self-made datasets was 99.61% and 95.84%, respectively. Similary, Xian et al. (in [19]) presented automatic metallic surface defect detection and recognition using a CNN. The authors designed a novel Cascaded Autoencoder (CASAE) architecture for segmenting and localizing defects. The segmentation results demonstrated an IoU score of 89.60%. Cheon et al. presented an Automatic Defect Classification (ADC) system for wafer surface defect classification and the detection of unknown defect class [20]. The proposed model adopted a single CNN model and achieved a classification accuracy of 96.2%. Finally, Civera et al. proposed video processing techniques for the contactless investigation of large oscillations to deal with geometric nonlinearities and light structures.

Though several works are available for narrow and enclosed space inspection applications using robot and computer vision algorithms, the defect detection and mapping of false ceilings are not yet widely studied. In the literature, very few works have reported robot-assisted ceiling inspection. Robert et al. in [21] introduced a fully autonomous industrial aerial robot using a top-mounted omni wheel drive system and an AR marker system. The proposed system can perform high precision localization and positioning to perform an ink-marker placement task for measuring and maintaining the ceiling. In [22], a flexible wall and ceiling climbing robot with six permanent magnetic wheels is proposed by Yuanming et al. to climb vertical walls and reach overhead ceilings. In [23], Ozgur et al. developed a 16-legged palm-sized climbing robot using flat bulk tacky elastomer adhesives. The proposed robot has a passive peeling mechanism for energy-efficient and vibration-free detachment to climb in any direction in 3D space. In [24], a self-reconfigurable false ceiling inspection robot is presented using an induction approach [25,26] and a rodent activity detection task [6]. A Perimeter-Following Controller (PFC) based on fuzzy logic was integrated into the robot to follow the perimeter of the false ceiling autonomously, and an AI-enabled remote monitoring system was proposed for rodent activity detection in false ceilings. All of these robots used for various purposes are summarized in Table 1. However, this research mainly focused on the robot design for various crawl spaces and does not involve the deterioration detection and mapping of false ceilings.

The literature survey indicates that there is a research gap in the robot-assisted false ceiling inspection field. Therefore, this work proposes a false ceiling inspection and deterioration mapping framework using a Deep-Learning (DL)-based deterioration detection algorithm and our in-house-developed teleoperated reconfigurable false ceiling inspection robot, known as the ‘Falcon’.

## 3. Overview of the Proposed System

**Figure 1:**
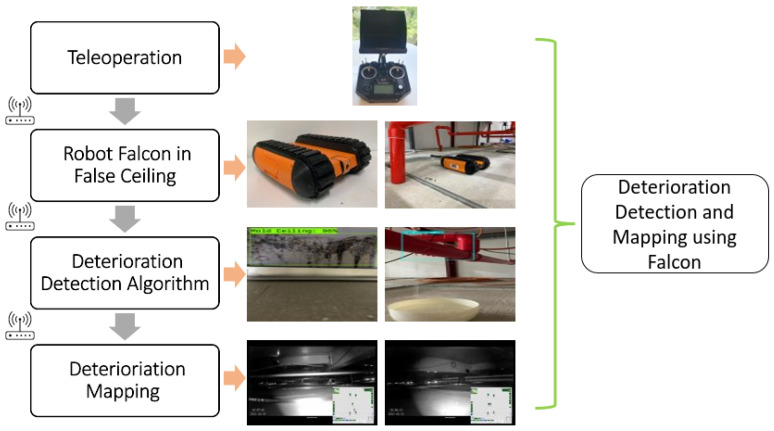
Overview of the proposed system.

Figure 1 shows an overview of the false ceiling inspection and deterioration mapping framework. Our in-house-developed crawl class Falcon robot was used for false ceiling inspection, and a deep-learning-based object detection algorithm was trained for deterioration detection from robot captured images. Further, a UWB localization module was used to localize the deterioration location and generate a deterioration map of a false ceiling. The detail of each module and functional integration is given as follows.

### 3.1. The Falcon Robot

**Figure 2:**
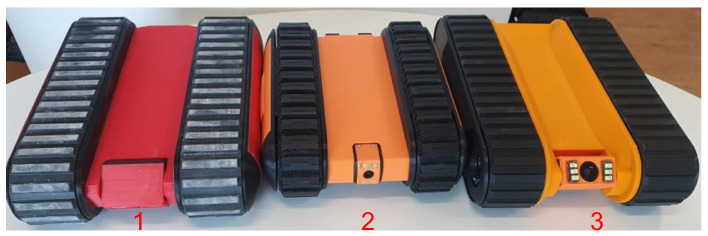
Different Version of Falcon Robot (Version 1, 2 and 3).

**Figure 3:**
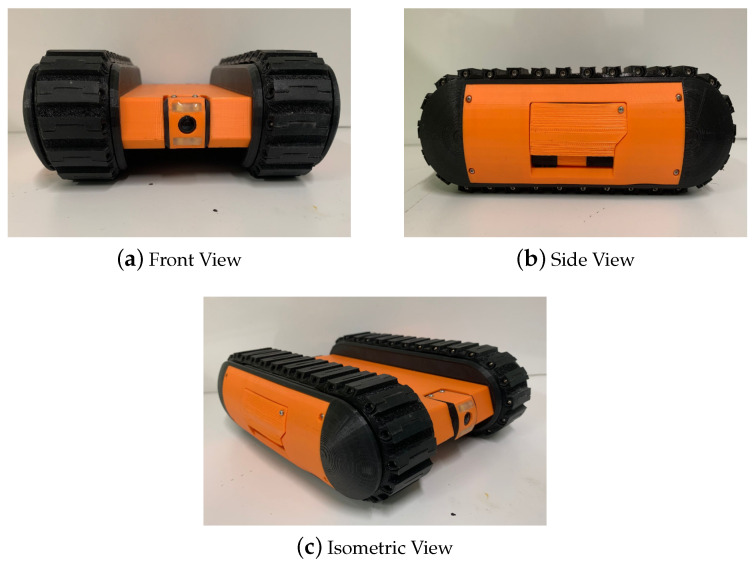
Different view of the Falcon Robot (Version 3).

A false ceiling panel is built using a fragile material such as Gypsum board or Plaster of Paris. Moreover, a false ceiling environment is crowded with components such as piping, electrical wiring, suspended cables, and protruding elements. Therefore, the Falcon was designed as a lightweight robot that can easily traverse obstacles. Furthermore, the camera used for image capturing or recording videos of the false ceiling environment is able to tilt the angle from 0 to 90 degrees for better accessibility in the crawled spaces. During the development stage, three versions of the Falcon robot were built due to changing requirements and design considerations, shown in Figure 2. In Version 2, the track mechanism is reinforced with a fork structure to avoid slippage while crossing obstacles in a false ceiling environment. Furthermore, a closed-form design approach was applied due to excessive dust-settling on electronic components. In Version 3 (as shown in Figure 3), a more precise IMU and more powerful motor is used. The robot height was further reduced to travel in spaces with an 80 mm height. All of the specifications of the Falcon robots are detailed in Table 2. The Falcon robot was powered with a 3 × 3.7 V, 3400 mAh battery that operates between 0.5 to 1.5 h with full autonomy functionalities. The operating range of the Falcon robot is directly determined by energy consumed by sensors and actuators, such as cameras, IMUs, cliffs, and motors. During autonomous operations, the motor operating at 1.6 A and 12 V consumes 33.2 W. Considering the battery power of 65.3 W, motors consume the highest fraction of the energy used for locomotion and to overcome tall obstacles. Further, the exploration tasks during false ceiling inspection also drain the energy, affecting the range of autonomy.

**Locomotion Module:** An important design consideration for a false ceiling robot is the form factor to overcome obstacles with a height of 55 mm and to traverse through low hanging spaces under 80 mm. In order to overcome these narrow spaces and tall obstacles, a locomotion module in the form of tracks that has maximized the contact area was used. The tracks extended along the dimension of the vehicle and were configured to be 236 (L) × 156 (W) × 72 (H) [mm × mm × mm]. The Falcon can operate regardless of the direction it flips over, as both sides of the locomotion modules are consolidated with hemispherical attachments to avoid stabilizing laterally. However, the operational terrain of the false ceiling may impose uncertainty on the Falcon robot. Therefore, the motors with higher specifications were chosen; e.g., a safety factor of 2 on a maximum inclined slope of 12 degrees.

**Control system:** A small-footprint, low-power ARM, Cortex-M7-powered, Teensy-embedded computing system was used as the onboard processor for the Falcon robot. It processes the velocity commands from the user and computes motor speeds using an inverse kinematic model. The MQTT server was employed to send the velocity command from the control station. In addition, the control unit is responsible for vital safety layer functionality to prevent the free-fall of the robots. Thus, the processor calibrates the IMU and cliff sensors to differentiate openings in the ceilings from the false noises while overcoming obstacles.

#### System Architecture

**Figure 4:**
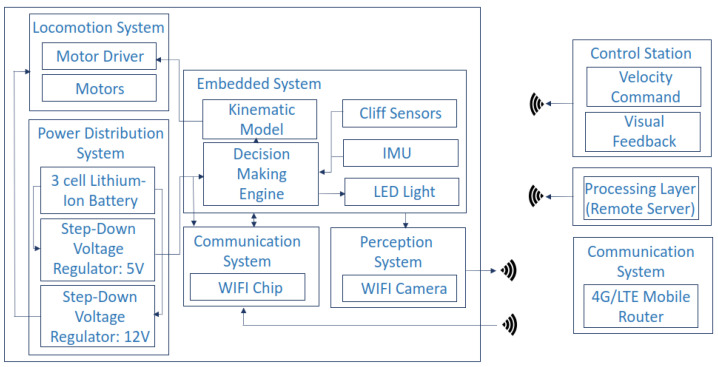
System architecture of the Falcon.

Figure 4 illustrates the system architecture of the Falcon robot. It consists of the following units: (1) a locomotion module, (2) a control unit, (3) a power distribution module, (4) a wireless communication module, and (5) a perception sensor.

**Perception Module** The WiFi camera module operates with a 5 V power rating and a 640×480 pixel density at 30 fps. The encoded video feed is a recorder and is additionally used to process the data through computer vision and machine learning algorithms to identify defects. Since the perception system relies heavily on lighting conditions, a NeoPixel stick with an 8∼50 RGB LED strip is used as the robot’s light source. Furthermore, a dedicated router is used to avoid data loss and for improved data security. Finally, a titling camera (up to 90 degrees) was incorporated considering broader field of view requirements using a servo motor controlled by A Teensy-embedded computing system.

### 3.2. Deterioration Detection Algorithm

**Figure 5:**
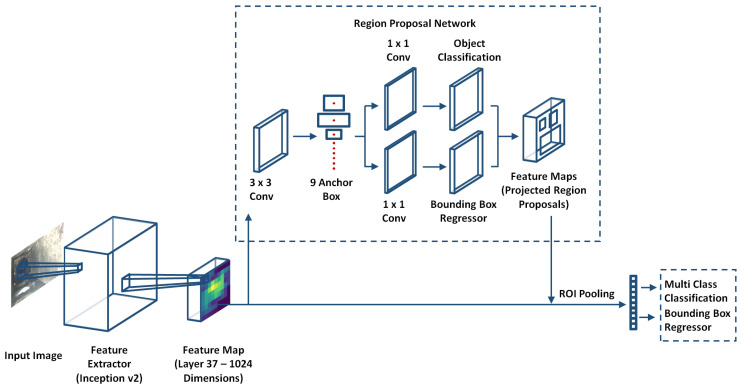
Functional block diagram of the deterioration detection algorithm.

Generally, deterioration factors in false ceiling environments are tiny and cover only a small number of the pixels of an image. Therefore, there is a requirement of a detection algorithm able to detect small objects to mitigate overlap or pixelated issues. Furthermore, the information extracted from images is lost due to multiple layers of the convolution neural network. The inspection algorithm needs an extensive, accurate, and apt framework with a small object detection capability. A Faster R-CNN model is an optimal framework when compared with similar CNN architectures and was used to detect small deterioration factors of the false ceiling environment in our case study [9,10]. Figure 5 shows an overview of the Faster R-CNN framework. Its architecture comprises three main components: the feature extractor network, the Region Proposal Network (RPN), and the detection network. All three components are briefly described in the following section.

#### 3.2.1. Feature Extractor Network

In our case study, Inception v2 performed the feature extraction task. It is an upgraded version of Inception v1, providing better accuracy and reducing computational complexity. Here, the input image size was 768×1024, and a total of 42 deep convolutional layers were used to build the feature extractor network. The number of feature maps directly controlled the task complexity, so an optimal 1024-size feature map (extracted from Layer 37 via a transfer learning scheme on a pre-trained dataset of COCO [27]) was fed into the Faster R-CNN. Further, in Inception v2, filter banks were expanded to reduce the loss of information, known as a ’representational bottleneck.’ Finally, the convolution 5×5 and 3×3 was factorized into two 3×3 convolutions and a combination of 1×3 and 3×1 convolutions, respectively, to boost the performance and reduce the computational cost. Further, Table 3 summarizes the layer details and input dimensions.

#### 3.2.2. Region Proposal Network

The Region Proposal Network (RPN) shares the output of the feature extractor network to the object detection and classification network. The RPN takes the feature map as an input (the output of the feature extractor network) and generates a bounding box with an objectness score using the anchor box technique first proposed by Shaoqing Ren et al. [28]. The anchor boxes are predefined, fixed-size boxes and detect objects of varying sizes and overlapping objects. It performs a 3×3 sliding window operation to generate anchor boxes in a 256-size feature map. Nine anchor boxes can be created from the combinations of sizes and ratios. Further, a stride of 8 (each kernel is offset by eight pixels from its predecessor) is used to determine the actual position of the anchor box in the original image. The output of the above convolution is fed into two parallel convolution layers, one for classification and the other for the boundary box regression. Finally, Non-Max Suppression (NMS) is applied to filter out the overlapping bounding boxes based on their objectness scores.

#### 3.2.3. Detection Network

The detection network consists of the Region of Interest (RoI) pooling layer and a fully connected layer. The shared feature map from the feature network and the object proposals generated by the RPN are fed into the RoI pooling layer to extract fixed-sized feature maps for each object proposal generated by the RPN. The fixed-sized feature maps are then fed two different fully connected layers with a softmax function. The first fully connected layer seeks to classify the object proposals into one of the object classes, plus a background class for removing bad proposals (N + 1 units, where N is the total number of object classes). The second fully connected layers seeks to better adjust the bounding box for the object proposal according to the predicted object class (4N units for a regression prediction of the x_center_, y_center_, width_center_, and height_center_ of each of the N possible object classes). Similar to the RPN, NMS is applied to filter out redundant bounding boxes and retain a final list of objects using a probability threshold and a limit on the number of objects for each class.

### 3.3. Deterioration Mapping

In our case study, the deterioration mapping function was accomplished using the UWB module. Explicitly, the UWB module was employed to track the mobile robot and localize the deterioration position. This location estimation feature was utilized and combined with the object detection module to identify, locate, and mark the deteriorations on the map of the false ceiling. At least three beacons must be installed where the actual number of beacons required is dependent on the complexity of the false ceiling infrastructure. In addition, sensor fusion was used to reduce localization errors and to calculate the exact position. It combines wheel odometry, IMU data, and UWB localization data to offer a more accurate location estimate. The beacon map was generated with the origin (0,0) as the location of the first beacon initiated and the relative position of other stationary beacons as landmarks. The mobile beacons within this relative map reflect the real-world location of the Falcon robot. As the robot explores and identifies deteriorations using the deterioration detection module, the location of the detected deterioration’s class is marked on the beacon map with their corresponding color code. It marks the deterioration’s class with an accuracy of a 30 cm radius on the map and is useful for the efficient inspection and maintenance of false ceilings.

### 3.4. Remote Console

The remote console is used to monitor and control the mobile Falcon robot for performing experiments. The primary mode of interaction happens via a transmitter and receiver system and directly in nature. The user controls the machine by sending signals that are transmitted through a remote. In our case study, the Taranis Q X7 from FrSky was used considering full telemetry capabilities as well as the RSSI signal strength feedback. The battery compartment uses two 18650 Li-Ion batteries and can be balance-charged via the Mini USB interface.

## 4. Experiments and Results

This section elaborates on the experimental setup and results of the proposed false ceiling deterioration detection and mapping framework. The experiments were carried out in five steps: dataset preparation, training and validation, prediction with a test dataset, a real-time field trial, and a comparison with other models.

### 4.1. Data-Set Preparation

The false ceiling deterioration training dataset was prepared by collecting images from various online sources and defect image dataset libraries (a surface defect database [29] and a crack image dataset [30,31]). In our dataset collection process, the common false ceiling deteriorations are categorized into four classes, namely, structural defects (spalling, cracks, and pitted surfaces), infestation (termites and rodents), electrical damage (frayed wires), and degradation in HVAC systems (molding, corrosion, and water leakage). Five thousand images were collected from an online source, and around 800 images were collected from a real false ceiling environment to train the deep learning algorithm. The CNN model was trained and tested using images with a 768×1024 pixel resolution. The “LabelImg” GUI was used for bounding boxes and class annotations. Annotations were recorded as XML files in the PASCAL Visual Object Classes (VOC) format.

**Figure 6:**
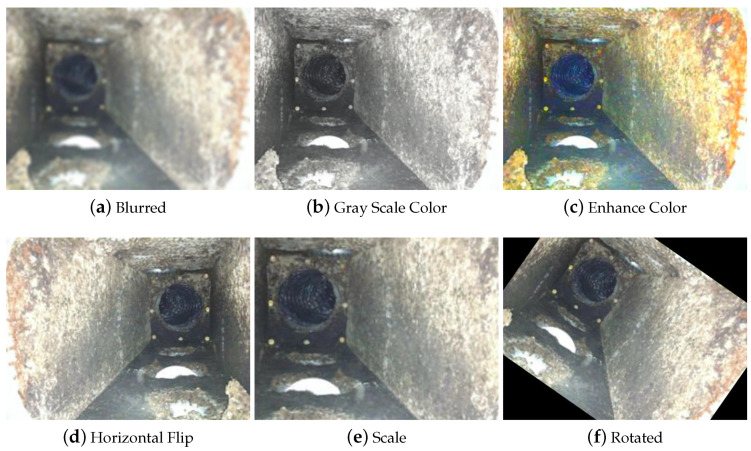
Sample of data augmentation of one image.

Further, the data augmentation process was applied on labeled images to help control the over-fitting and class imbalance issues in the model training stage. Data augmentation processes such as horizontal flips, scaling, cropping, rotations of the image, blurring, grayscale colors, and color enhancing were applied to the collected images. Figure 6 shows a sample of the data augmentation of one image. Further, Table 4 elaborates the settings of the various types of augmentations applied.

### 4.2. Training and Validation

The object detection model, the Faster R-CNN, was built using the TensorFlow (v1.15) API and the Keras wrapper library. The pre-trained Inception V2 model was used as a feature extraction module. It was trained on the COCO dataset. A Stochastic Gradient Descent (SGD) optimizer was used for the training of the Faster R-CNN module. The hyper-parameters used were 0.9 for momentum, an initial learning rate of 0.0002, which decays over time, and a batch size of 1.

The model was trained and tested on the Lenovo ThinkStation P510. It consists of an Intel Xeon E5-1630V4 CPU running at 3.7 GHz, 64 GB of Random Access Memory (RAM), and a Nvidia Quadro P4000 GPU (1792 Nvidia CUDA Cores and 8 GB GDDR5 memory size running at a 192.3 GBps bandwidth). The same hardware is used to run as a local server to allow the Falcon robot to carry out inference during real-time testing.

The K-fold (here K = 10) cross-validation technique was used for validating the dataset and model training accuracy. In this evaluation, the dataset was divided into K subsets, and K−1 subsets were used for training. The remaining subset was used for evaluating the performance. This process was run K times to obtain the mean accuracy and other quality metrics of the detection model. K-fold cross-validation was performed to verify that the images reported were accurate and not biased towards a specific dataset split. The images shown were attained from the model with good precision. In this analysis, the model scored a 91.5% mean accuracy for k = 10. This indicates that the model is not biased towards a specific dataset split.

### 4.3. Prediction with the Test Dataset

**Figure 7:**
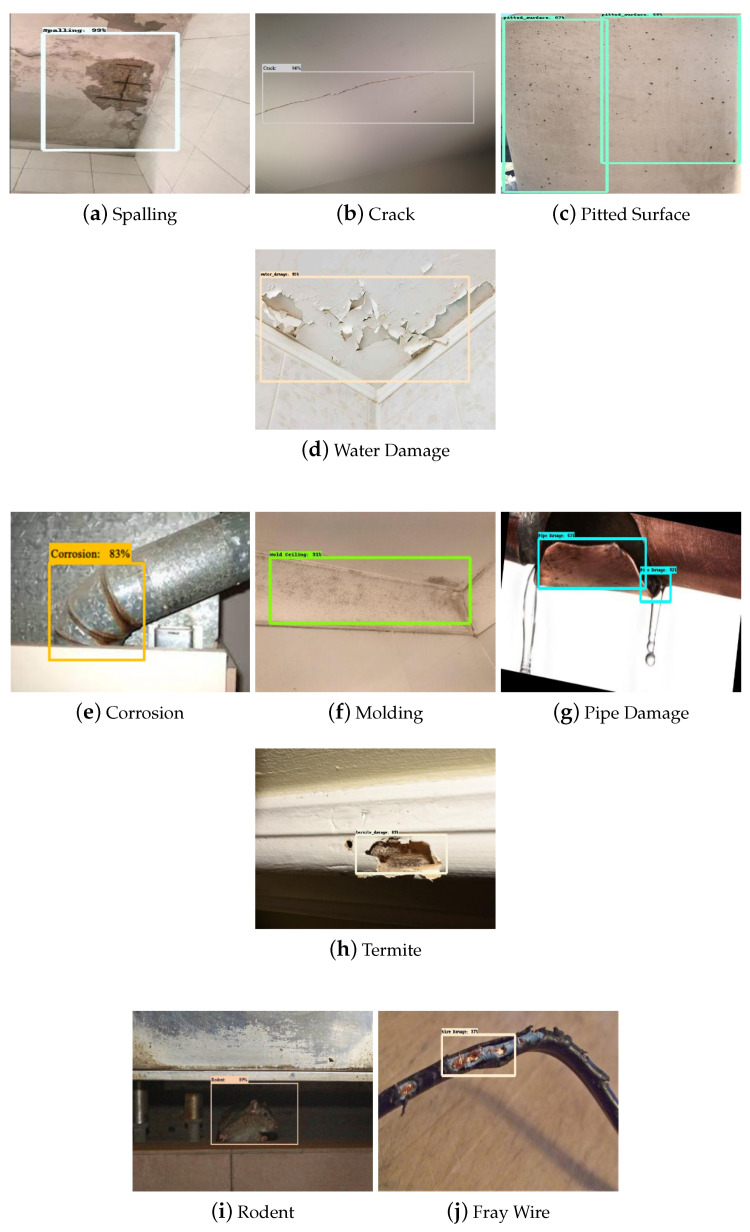
Structural Defects (**a**–**d**), degradation in HVAC system (**e**–**g**), infestation (**h**,**i**), electrical damage (**j**) during Offline Testing.

The trained model’s deterioration detection and classification accuracy were evaluated using the test dataset. In this evaluation process, 100 images were tested from each class. These test datasets were not used in the training and cross-validation of the model. Figure 7 shows the detection results of the given test dataset.

The experimental results show that the deterioration detection algorithm accurately detected and classified the deterioration in the given test images with a high confidence level average of 88%. Further, the model classification accuracy was evaluated using standard statistical metrics such as accuracy (Equation (1)), precision (Equation (2)), recall (Equation (3)), and $F_{m}easure$ (Equation (4)).

(1) $$Accuracy\left(Acc\right)=\frac{tp+tn}{tp+fp+tn+fn}$$

(2) $$Precision\left(Prec\right)=\frac{tp}{tp+fp}$$

(3) $$Recall\left(Rec\right)=\frac{tp}{tp+fn}$$

(4) $$F_{measure}\left(F_{1}\right)=\frac{2\times precision\times recall}{precision+recall}$$

**Figure 8:**
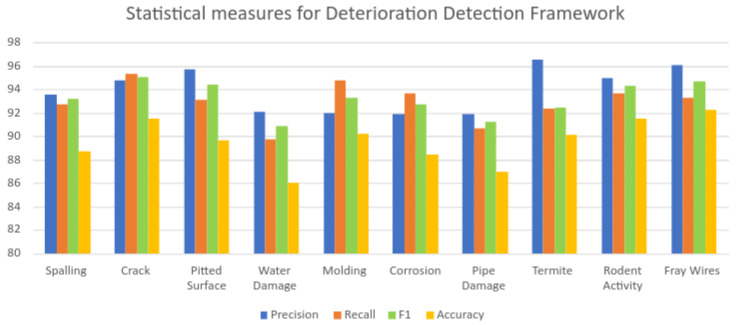
Graphical representation of the statistical measures of the proposed framework.

Here, tp,fp,tn,fn represent the true positives, false positives, true negatives, and false negatives, respectively, as per the standard confusion matrix. Table 5 provides the statistical measure results of the offline test. Figure 8 demonstrates the graphical representation of Table 5 for improved visualization.

The statistical measures experimental result indicate that the proposed framework detected structural defects with an average accuracy of 88.9%, degradation in the HVAC system at an 88.56% accuracy, infestation at a 90.75% accuracy, and electrical damage at a 92.2 % accuracy.

### 4.4. Real-Time Field Trial

The real-time field trial experiments were performed in two different false ceiling environments, including the Oceania Robotics prototype false ceiling testbed and the SUTD ROAR laboratory real false ceiling. The false ceiling testbed consists of frames, dividers, pipes, and other common false ceiling elements. For experimental purposes, various deteriorations in false ceilings such as frayed wire, damaged pipes, and termite damage were manually created and placed in the prototype environment. Some of the defects, such as pitted surfaces and spalling, were fabricated using printed images of these defects. These printed images were glued at various locations in a false ceiling testbed for experimental purposes. Further, to track the robot position and identify the false ceiling deterioration location, a mobile beacon was placed on the top of the Falcon, and stationary beacons were mounted on projecting beams or sidewalls. The mobile beacons were the transmitters operating in unique frequencies, while all of the stationary beacons operated in the same frequency and behaved as receivers. The location of the moving beacons was calculated based on triangulating the distances from stationary beacons, and the current location was updated at a frequency of 16 Hz. With an accuracy of up to 2 cm and a bandwidth accommodating up to six mobile devices seamlessly, the beacon system implemented was used for false ceiling deterioration mapping and localization.

**Figure 9:**
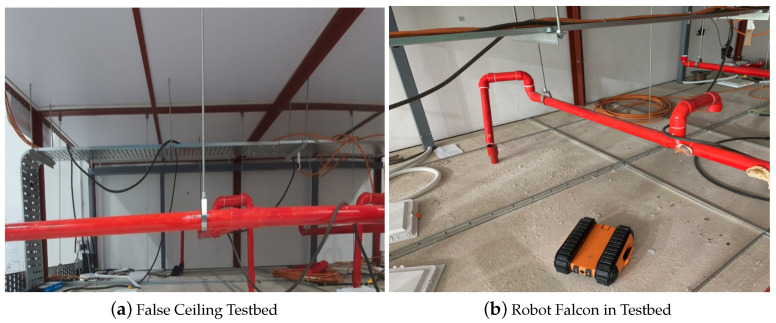
Falcon and the false ceiling testbed prototype.

**Figure 10:**
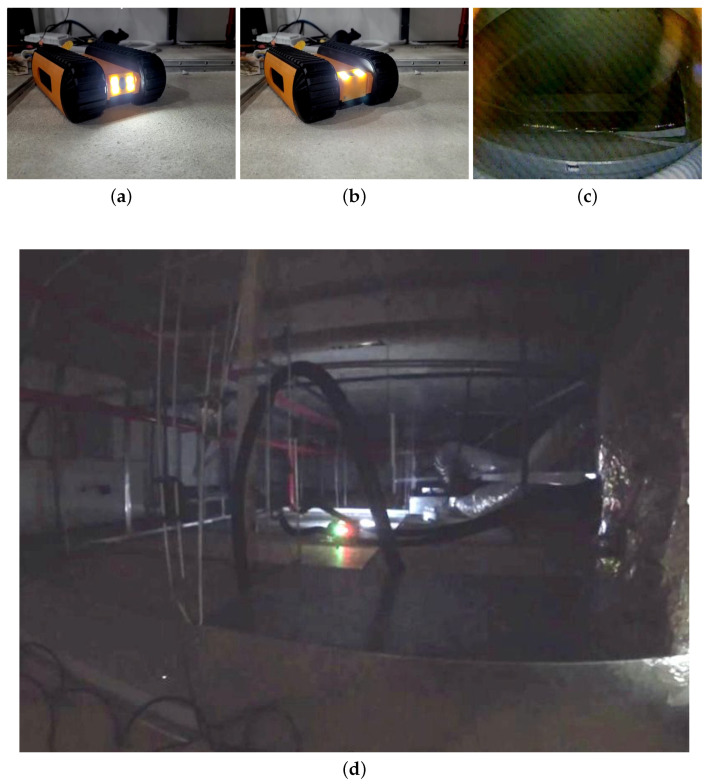
Falcon’s performance on the false ceiling prototype at Oceanica Robotics. (**a**) Falcon in Prototype of False Ceiling (Camera at 0 degree); (**b**) Falcon in Prototype of False Ceiling (Camera at 90 degree); (**c**) Image collected by Falcon; (**d**) Falcon in Prototype of False Ceiling (Zoomed out).

**Figure 11:**
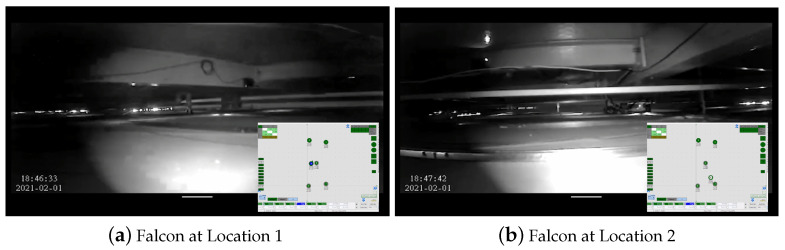
Falcon’s performance on the false ceiling at the SUTD ROAR Laboratory.

Figure 9 and Figure 10 show the Falcon robot in the prototype of the false ceiling (Oceania Robotics test bed), while Figure 11 shows the robot in a real false ceiling environment (SUTD ROAR Laboratory). During the inspection, the robot was controlled by a mobile GUI interface, and the robot’s position and the defect region were localized through UWB modules fixed in the false ceiling environment. The robot was paused at each stage for a few seconds to capture better quality images in these real-time field experiments.

**Figure 12:**
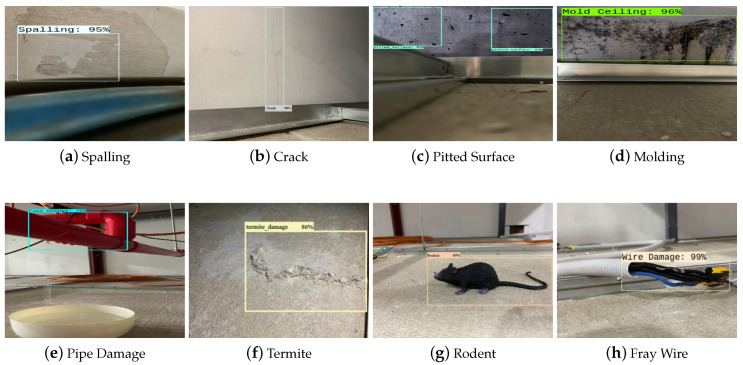
Structural defects (**a**–**c**), degradation in the HVAC system (**d**,**e**), infestation (**f**,**g**), and electrical damage (**h**) during online testing.

**Figure 13:**
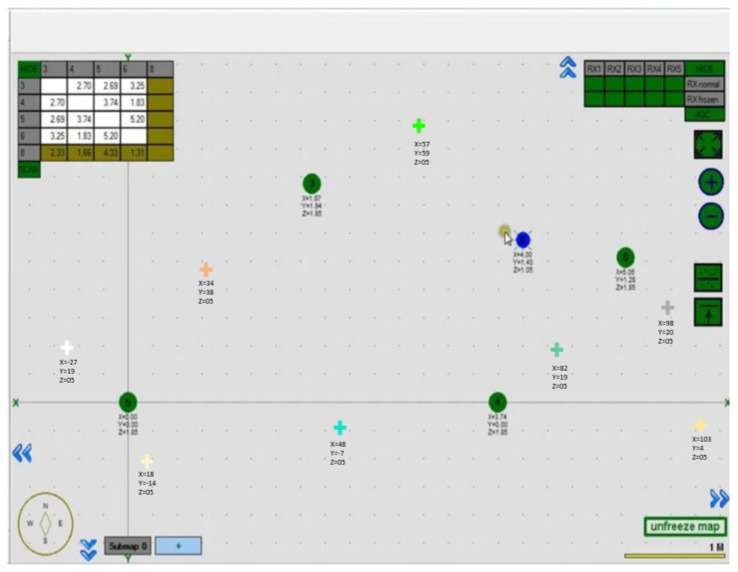
Beacon maps with static beacons and a mobile beacon.

The captured images were transferred to a high-powered GPU-enabled local server for false inspection tasks via WiFi communication. Figure 12 depicts the real-time filed trial deterioration detection results of the false ceiling testbed, and its localization results are shown in Figure 13. These deterioration-detected image frame locations were identified by fusing the beacon coordinates, wheel decoder data, and IMU sensor data on the Marvel Mind Dashboard tracking software. Figure 13 also shows the deterioration location mapping results for the real-time field trials, where the color codes indicate the class of deterioration.

The findings of the experiment reveal that the Falcon robot’s maneuverability was stable. It could move around a complex false ceiling environment and accurately capture it for false ceiling deterioration identification. The detection algorithm detected most of the false ceiling deterioration in the real-time field trial with a good confidence level and scored an 88% mean detection accuracy. Furthermore, the Falcon robot’s position on the false ceiling could be reliably tracked using the UWB localization results. This will further help inspection teams to identify defects and degradation efficiently.

## 5. Discussion

**Figure 14:**
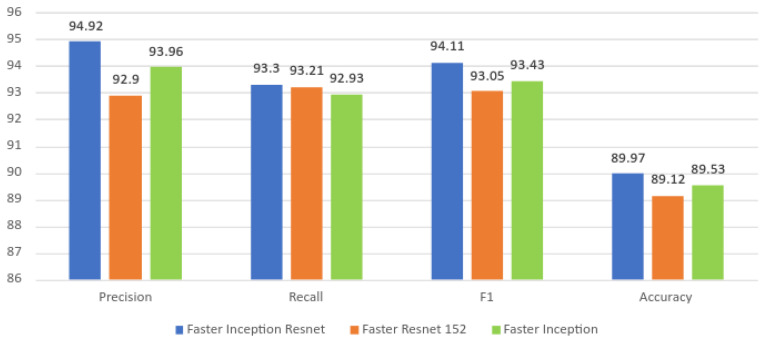
Graphical representation of the comparison with other detection frameworks.

The proposed system’s performance is discussed in this section by a comparison with two models (Faster Inception ResNet and Faster Resnet 152) and other existing studies. The comparison analysis findings are shown in Table 6, Table 7 and Table 8. The three detection frameworks were trained on the same image dataset and with the same number of epochs. Here, overall detection accuracies of 86.8% for the Faster Resnet 152 and 86.53% for the Faster Inception Resnet were observed. The detection accuracy of these two models was relatively low due to a high false-positive rate and misclassification issues due to similar deterioration factors and the impact of object illumination. These issues can be further resolved by retraining the algorithm with misclassified classes and applying nonlinear detrending techniques [32]. Further, Figure 14 shows a graphical representation of Table 8 for improved visualisation.

The cost of training and testing is shown in Table 9. In that analysis, we found that the proposed model also had a lower execution time compared to the Faster Inception Resnet and Faster Resnet 152 models. Because of this, the framework that has been proposed is better suited for false ceiling deterioration detection tasks.

Table 10 shows the accuracy of various defect detection algorithms based on different classes. However, a fair comparison is lacking because their algorithm, datasets, and training parameters are not the same. Finally, the proposed method involves robotic inspection, which is another contribution with respect to the state of the art.

## 6. Conclusions

False ceiling defect detection and mapping were presented using our in-house-developed Falcon robot and the Faster Inception object detection algorithm. The efficiency of the proposed system was tested through a robot maneuverability test and showed defect detection accuracy in offline and real-time field trials. The robot’s maneuverability was tested in two different false ceiling environments: the Oceania Robotics prototype false ceiling testbed and the SUTD ROAR laboratory real false ceiling. The experimental results proved that the Falcon robot’s maneuverability was stable and that its defect mapping was accurate in a complex false ceiling environment. Further, the defect detection algorithm was tested on a test dataset, and real-time false ceiling images were collected by the Falcon robot. The experimental results show that Faster Inception has a good trade-off between detection accuracy and computation time, with a detection accuracy of 89.53% for detecting deterioration in real-time Falcon-collected false-ceiling-environment video streams, whereas the average detection accuracies of Faster Resnet 152 and Faster Inception Resnet were 86.8% and 86.53%, respectively. Further, Faster Inception required only 68 ms to process one image on the local server, which is lower compared with other algorithms, including Faster Inception Resnet and Faster Resnet 152. Further, the mapping results precisely indicated the location of deterioration on the false ceiling. Thus, it can be concluded that the suggested method is more suited for defect detection in false ceiling environments and can improve inspection services. In our future work, we plan to add more features to the false ceiling inspection framework, such as olfactory contamination detection.

## Figures and Tables

**Table 1:** Summary of research.

Reference	Typology of the Platform	Aim
Gary et al. [5]	Wheeled Robot	Detect common features of underfloor void
Balakrishnan et al. [6]	Tracked Robot	Rodent Activity Monitoring
Protopapadakis et al. [7]	Wheeled Robot	Crack detection for tunnel inspection
Menendez et al. [8]	Wheeled Robot	Tunnel structural inspection
Palanisamy et al. [9]	Wheeled Robot	Drain Structural Defect Detection
Melvin et al. [10]	Wheeled Robot	Drain blockage inspection
Kouzehgar et al. [11]	Wheeled Robot	Automatic glass crack detection
Hung et al. [15]	Wheeled Robot	Inspection of steel structures and bridges
Gui et al. [16]	Wheeled Robot	Airport runway inspection
Ladig et al. [21]	Aerial Robot	Platform to do high precision localization and positioning
Ozgur et al. [23]	Sixteen-legged climbing robot	To climb in any direction in 3D space
Hayat et al. [24]	Reconfigurable wheeled robot	False-ceiling inspection

**Table 2:** Technical specifications of the Falcon robot.

Description	Specification
Dimension [L × W × H]	0.236 m × 0.156 m × 0.072 m
Weight (including battery)	1.3 kg
Type of Locomotion Drive	Track
Top & Bottom Ground Clearance	0.011 m, 0.011 m
Operating Speed	0.1 m/s
Maximum Obstacle Height	0.055 m
Operational Duration	0.5 h–0.75 h
Battery	3-cell Lithium Ion
Operation Mode	Teleoperation (with integrated sensors to detect falls and stops autonomously)
Communication Mode	Wi-Fi through a local MQTT server
Camera Specifications (onboard light source)	VGA 640 × 480, up to 30 fps, 60 degree view angle, 20 cm-infinity focusing range

**Table 3:** Inception v2 backbone.

Layer Details	Input Dimensions
Conv	299×299×3
Conv	149×149×32
Conv	147×147×32
Pool	147×147×64
Conv	73×73×64
Conv	71×71×80
Conv	35×35×192
3× Inception module A	35×35×288
5× Inception module B	17×17×768
2× Inception module C	8×8×1280
Pool	8×8×2048
Linear	1×1×2048
Softmax	1×1×1000

**Table 4:** Augmentation types and settings.

Augmentation Type	Augmentation Setting
Blurring	gaussianblur (from sigma 1.0× to 3.0×)
Grayscale	individual rgb spectrum (from factor 0 to 1.5×)
Color Enhancing	contrast (from 0.5× to 1.5×)
Horizontal Flip	flip the image horizontally
Scaling	0.5× to 1.5×
Rotation	from −45 degree to +45 degree
Translation	X-axis (−0.3× to 0.3×) Y-axis (−0.3× to 0.3×)

**Table 5:** Statistical measures for the deterioration detection framework (the proposed framework).

Category	Class	Precision	Recall	F1	Accuracy
Structural Defect	Spalling	93.60	92.77	93.16	88.70
	Crack	94.77	95.32	95.04	91.50
	Pitted Surface	95.70	93.13	94.39	89.70
	Water Damage	92.11	89.74	90.91	86.00
Degradation in HVAC	Molding	91.95	94.74	93.32	90.20
	Corrosion	91.88	93.63	92.74	88.50
	Pipe Damage	91.89	90.67	91.28	87.00
Infestation	Termite	96.59	92.39	94.44	90.10
	Rodent Activity	95.01	93.63	94.31	91.52
Electrical Damage	Fray wires	96.13	93.29	94.69	92.23

**Table 6:** Statistical measures for the deterioration detection framework (Faster Resnet 152).

Category	Class	Precision	Recall	F1	Accuracy
Structural Defect	Spalling	87.12	86.11	86.61	86.4
	Crack	90.87	90.47	90.67	90.2
	Pitted Surface	85.61	84.8	85.2	84.2
	Water Damage	86.79	87	86.89	84.6
Degradation in HVAC	Molding	90.69	91.09	90.89	89.8
	Corrosion	88.86	89.27	89.07	87.6
	Pipe Damage	85.51	84.89	85.2	83.4
Infestation	Termite	85.55	86.56	86.05	84.8
	Rodent Activity	90.38	88.99	89.68	90.8
Electrical Damage	Fray wires	90.54	88.86	89.7	86.2

**Table 7:** Statistical measures for the deterioration detection framework (Faster Inception Resnet).

Category	Class	Precision	Recall	F1	Accuracy
Structural Defect	Spalling	88.19	88.81	88.5	85.8
	Crack	92.51	92.92	92.72	90.4
	Pitted Surface	86.57	85.95	86.26	83.9
	Water Damage	87.8	86.97	87.38	84.3
Degradation in HVAC	Molding	92.05	92.46	92.27	90.2
	Corrosion	88.84	87.61	88.22	87.2
	Pipe Damage	86.76	87.17	86.97	84.1
Infestation	Termite	85.16	84.13	84.64	83.2
	Rodent Activity	93.16	92.54	92.85	91.1
Electrical Damage	Fray wires	87.21	88.03	87.62	85.1

**Table 8:** Comparison with other object detection frameworks.

Class	Precision	Recall	F1	Overall Accuracy
Faster Inception Resnet	94.92	93.3	94.11	86.5
Faster Resnet 152	92.9	93.21	93.05	86.8
Faster Inception (Proposed Model)	93.96	92.93	93.43	89.53

**Table 9:** Computational cost analysis.

Algorithm	Training Time (Hours:Minutes)	Speed (Milliseconds)
Faster Inception Resnet	23:20	647
Faster Resnet 152	19:48	135
Faster Inception (Proposed Model)	16:18	68

**Table 10:** Comparison of results with other methodologies in related work.

Reference	Algorithm	Accuracy
Gary et al. [5]	Mask-RCNN	80.00%
Hung La et al. [15]	CNN-based image stitching and 3D registration	93.10%
Gui et al. [16]	CNN-based	70.00%
Perez et al. [17]	VGG-16	87.50%
Xing et al. [18]	CNN model (SCN) and optimised IoU	85.84%
Xian et al. [19]	Cascaded encoder (CASAE)	89.60%
Cheon et al. [20]	Single CNN	96.20%
Proposed Method	Faster R-CNN Inception	89.53%

## Data Availability

Not applicable.
